# Neuropsychiatric and Psychosocial Issues of Patients With Hepatitis C Infection: A Selective Literature Review

**DOI:** 10.5812/hepatmon.8340

**Published:** 2013-01-07

**Authors:** Amirhossein Modabbernia, Hossein Poustchi, Reza Malekzadeh

**Affiliations:** 1 Digestive Disease Research Centre, Shariati Hospital, Tehran University of Medical Sciences, Tehran, IR Iran; 2 Psychiatric Research Centre, Roozbeh Hospital, Tehran University of Medical Sciences, Tehran, IR Iran

**Keywords:** Anxiety, Depression, Fatigue, Hepatitis C, Mental Health, Neurobehavioral Manifestations, Social Stigma

## Abstract

**Context:**

We briefly reviewed the evidence on the association of hepatitis C (HCV) infection with several aspects of mental and psychosocial health.

**Evidence Acquisition:**

Medline was searched with appropriate keywords. The primary sources were the systematic reviews. If systematic reviews were not available for a subject, then the most relevant and methodologically sound original studies were selected.

**Results:**

HCV infection is associated with poorer health-related quality of life, and physical, mental, and social health. A part of impaired health of these patients is related to cirrhosis, intravenous drug use, co morbid psychiatric disorders, stigmatization, poor social support, alcohol abuse, and interferon treatment. However, HCV itself is also associated with poorer health status particularly in the physical and cognitive domains, which might be related to brain alterations induced by the virus. Interferon treatment is an important cause of depression in HCV patients and sometimes is associated with irritability, manic episode, or acute confusional state. Social health of HCV patients is significantly impaired by stigmatization, poor social support, psychiatric comorbidties, and impaired coping. Psychosocial impairment of HCV patients significantly impairs their treatment adherence. A supportive and nonjudgmental multidisciplinary team is required for optimal management of these patients.

**Conclusions:**

Patients with HCV infection had complex neuropsychiatric and psychosocial problems. These problems are challenges for management of HCV infection, affect the patient’s care significantly, and might alter the course of the disease. A multidisciplinary approach, a supportive environment, and a nonjudgmental healthcare team are required for optimal medical and psychosocial management of patients with HCV.

## 1. Context

In the present paper, we briefly review the essential evidence on the association of HCV infection with several aspects of mental and psychosocial health of the patients. This review primarily aimed to inform the clinicians and the researchers about this broad and important area of management of patients with HCV. For those readers who are interested in the details of each problem appropriate references are introduced.

## 2. Evidence Acquisition

Medline was searched with keywords: HCV, hepatitis C, psychological, psychosocial, quality of life, depression, anxiety, fatigue, anger, irritability, mental health, physical health, cognition, cognitive, stigma, psychiatric, psychiatry, mood, behavior, addiction, habits, coping, stress, social support, pain, fibromyalgia. The primary sources for the review were systematic reviews. If systematic reviews were not available for a subject, the most relevant and methodologically sound original studies were selected.

## 3. Results

Hepatitis C virus (HCV), an RNA-virus which primarily infects liver parenchyma is currently a major health problem (1, 2). It is associated with chronic liver disease, cirrhosis, and hepatocellular carcinoma and imposes a huge burden on the health care programs and the society. In addition to its impact on liver, patients with HCV encounter problems in a wide variety of health areas including health related quality of life (HRQOL), and mental and physical health (3, 4). Importantly, to reduce the imposed burden of the disease, an important step is to evaluate and manage these factors in an appropriate manner. While some of these health problems are attributable to the factors which generally accompany HCV (such as addiction or personality problems), a growing body of evidence suggest that they might be related to the HCV itself.

### 3.1. HRQOL

HCV negatively affects the HRQOL of patients (5). A systematic review of 32 studies assessed the impact of HCV infection on HRQOL. Analysis of 15 studies comparing HCV patients versus healthy controls indicated diminished HRQOL in HCV patients with a moderate to large effect size. Furthermore, it was found that HCV most prominently affected social and physical functioning, general health and vitality. The review also showed that HRQOL was better in patients who achieved sustained virological response (SVR) than in those who did not, with more impact on the general health and role-physical subscales. The study also suggested that small histopathological or biochemical changes were unlikely to affect HRQOL. However, cirrhosis significantly diminished HRQOL of the patients with HCV (6). A recent systematic review of 66 studies provided rather consistent evidence of reduced HRQOL in untreated HCV patients (7). Results of the same review demonstrated reduced HRQOL during the treatment and improved HRQOL post-treatment. Improved post-treatment outcomes were particularly prominent in the patients with SVR. In addition to the impact of treatment, several other factors affect HRQOL in HCV-infected patients. Several studies have found that a part of the reduction in HRQOL can be explained by substance addiction, cirrhosis, and other comorbidities (8). However, there is a strong line of evidence that HRQOL is impaired in HCV patients even when these factors are controlled (9-11). HCV seems to differ from its hepatitis B (HBV) counterpart in affecting the health of the individual by more profound effect on HRQOL, and mental and physical health (1, 9-15). In a recent study comparing HRQOL between HBV and HCV patients after controlling several related factors such as addiction, alcohol, severity of liver disease, etc. the authors found strong evidence for the relationship of HCV infection with impaired physical health (9). Importantly, in a separate study the same authors also found inverse association between the levels of brain-derived neurotropic factor (BDNF) to physical health in patients with HCV but not HBV infection (11). BDNF is a neurtophic factor which is produced by several body tissues (most importantly brain) and is important in neuronal development and differentiation. Mentioned findings together with the evidence of cognitive dysfunction and brain involvement in HCV strongly raise the possibility that health problems in these patients are associated with direct effect of HCV on brain (11, 14-19). A review of the studies on the effect of liver transplantation (OLT) on HRQOL in patients with HCV concluded that OLT generally improved the HRQOL scores in these patients, particularly in social functioning and physical health domains. However, these patients still had significantly poorer HRQOL (particularly in psychological domains) than healthy controls. Indeed, OLT had little effect on long-term psychological improvement. This was in contrast to pre-OLT state in which the psychological scores (but not other domains of health) of the patients were closer to the general population. Importantly, knowledge of the HCV recurrence significantly reduced the HRQOL in patients following OLT (20).

### 3.2. Fatigue and Psychosomatic Symptoms

Fatigue is probably the most common extra hepatic manifestation of HCV infection with a prevalence of around 50% (21). Most studies have shown higher fatigue levels in the patients with HCV compared with patients with HBV infection and healthy controls (22). The impaired physical health of patients with HCV has been replicated using a wide variety of HRQOL and fatigue measures. Chronic fatigue syndrome (CFS) is unrelated to HCV infection and is generally severer than the fatigue experienced by HCV-infected patients (23). Fatigue in HCV is of central type and is associated with cognitive impairment and depression. Psychological and psychiatric comorbidities as well as old age, being female and single have been important predictors of fatigue in several studies (9, 24, 25). Several biological markers including low serum carnitine, creatinine, and BDNF concentrations (9, 11, 26), and higher serum leptin concentrations (27) also predicted higher fatigue levels in HCV-infected patients. Other biochemical, pathological, and viral parameters are unrelated to fatigue (9, 21, 27, 28). An important correlate of fatigue in HCV-infected individuals is HCV itself (9, 11). Fatigue was substantially improved following SVR after antiviral treatment (28). IFN-induced fatigue in HCV-infected individuals is associated with some molecular signatures of inflammatory pathways such as cAMP responsive element binding protein/activation transcription factor (CREB/ATF) and p38 mitogen activated protein kinase (MAPK) (28-30). Several treatment options such as ondansetron, exercise, modafinil, armodafinil, amantadine and acetyl L-carnitine have been used successfully for management of HCV-induced fatigue (22, 28-34).Other physical symptoms associated with HCV infection are arthralgia, fibromyalgia, myalgia, and chronic pain which together have been reported in more than half of the patients in some series (21, 24, 35-39).

### 3.3. Psychiatric Disorders

HCV is one of the few infections, in addition to HIV, which is heavily linked to psychiatric disorders (40, 41).A cause for this strong association is that illicit drug injection (IDU) is the most important risk factor for HCV infection. Drug injection is common in the patients with personality problems, besides other high risk behaviors (alcoholism, sexual high risk behaviors), and mood disorders (42). Even among non-injection drug users, up to 30% might be infected with HCV (43). Among all abused substances alcohol is the only one that can boost the progression of the liver involvement and therefore should be taken very seriously. United States veterans have been most widely studied regarding the prevalence of trimorbidity (HCV, substance abuse and psychiatric illness). In the largest study on veterans, 85% of 33842 tested individuals had evidence of past or present psychiatric disorders or substance abuse, and more than 60% of them had comorbid substance abuse and psychiatric disorders (44). However, in non-veteran patients the rates of psychiatric disorders have been much lower (45, 46). Alcoholism is associated with higher prevalence of anti-HCV antibody positivity. Alcohol acts synergistically with HCV to deteriorate the liver disease and reduces the treatment response to Interferon (IFN) primarily by decreasing the compliance (47). IFN-alpha is increasingly being used for HCV treatment. Besides its beneficial effects, IFN alpha may induce a variety of neuropsychiatric side effects such as acute confusional state, depressive syndrome, and agitated manic episode (48). Following IFN treatment of patients with HCV, up to 70% may develop depression (41). Several mechanisms have been proposed for this association including altered monoamine metabolism (41), increased rate of apoptosis (49), BDNF reduction (50), and altered hypothalamus-pituitary-adrenal axis function (48). In a meta-analysis of 26 observational studies, the authors concluded that one fourth of patients who underwent treatment with interferon and ribavirin, developed major depressive disorder (MDD). High baseline serum interleukin 6 concentrations, being female, history of psychiatric disorder, sub-threshold depressive symptoms, and low educational level significantly predicted the occurrence of MDD during antiviral treatment (51). Neuro-vegetative/somatic symptoms of depression occur early in the course of IFN therapy, whereas cognitive/mood symptoms often become evident after the fourth week of treatment (41). Depression, anxiety, and cognitive complaints are responsive to serotonergic antidepressants, whereas neuro-vegetative symptoms such as decreased appetite, fatigue, sexual impairment, and psychosomatic symptoms are less responsive to SSRIs (52). Neurovegetative symptoms can be better managed with serotonin-norepinephrine reuptake inhibitors, bupropion, methylphenidate or modafinil (48). In a systematic review of 64 observational and interventional studies, the authors showed that SSRIs might be the first choice for treatment of interferon-induced MDD (53). IFN alpha-induced acute confusional states present with psychomotor retardation, disorientation, Parkinsonism, and psychosis. IFN alpha is also capable of inducing manic symptoms. Severe mania which can occur as a result of IFN administration should be treated with immediate discontinuation of IFN and antidepressants and initiation of a mood stabilizer (48). A recent retrospective study on 910 patients with HCV compared the rate of psychiatric complications during the treatment with IFN between patients with bipolar disorder, patients with MDD, and patients without psychiatric illness. Psychiatric complications occurred in more than half of the patients with MDD or bipolar disorder and one third of the patients without psychiatric illness. However manic episode occurred only in one out of 38 patients with bipolar disorder. Therefore, it was concluded that patients with bipolar disorder who were selected carefully can achieve successful treatment outcomes comparable to those in patients without bipolar disorder (54). Specific risk factors for IFN-induced suicide are unknown yet (41) . European Expert Consensus Statement has recently released a guideline for management of mental health disturbances associated with IFN treatment. According to the Consensus, before starting the antiviral treatment, a complete psychiatric history should be taken, and information regarding psychiatric adverse effects should be given. Following initiation of the antiviral treatment, mood status should be assessed every four weeks in the first three months of treatment and then at least every 12 weeks after the end of the treatment. The intervals between two monitoring visits should be halved in the case of psychiatric comorbidity or substance abuse. Patients with psychiatric comorbidity or substance abuse do not generally differ in their treatment outcomes from other patients. However, the Consensus suggested acute and major psychiatric disorders and acute ongoing and uncontrolled IV drug abuse as relative contraindications to antiviral treatment. The Consensus recommended citalopram as firs-line treatment for IFN-induced depression. Antidepressant treatment should be continued for at least 12 weeks following the end of IFN treatment. Antidepressant treatment is also indicated for those patients with baseline depressive symptoms and for those with history of IFN-induced depression. Treatment with antidepressants is generally not recommended for all HCV patients and should be used on an individual basis (41). Not only IFN itself is associated with depression, but it seems that HCV might also be associated with mood problems. Again, a part of this link can be explained by HCV-accompanied factors (such as personality problems, high-risk behavior, stigma, and substance abuse). However, there is evidence that some specific genotypes of HCV such as 3a might be associated with increased risk of depression (9, 55). Evidence of HCV neuro invasion may be another explanation for the association between mental disorders and HCV (9, 55).Anxiety disorders in patients with HCV seem to parallel depressive disorders in prevalence rates. Using Structured Clinical Interview for DSM-IV Axis I (SCID-I) in a sample of 500 patients with HCV, prevalence of any depressive disorder, MDD, generalized anxiety disorders, and panic disorders were 18.2%, 6.4%, 7.0% and 5.8% respectively (56).

### 3.4. Sleep Problems

In a review, Sockalingam et al. (57) addressed the sleep disturbances in HCV. Around 60% of patients with HCV may suffer from sleep disturbances due to various reasons including comorbid psychiatric disorders, substance abuse, and advanced liver disease. There is evidence for increased rate of sleep disruption, obstructive sleep apnea, and probably restless leg syndrome in patients with HCV. One fifth to one third of the individuals being treated with pegylated or conventional IFN experience sleep problems. The mechanisms proposed for IFN-induced sleep disturbances are inflammatory cytokines, altered serotonin metabolism, and modulation of narcolepsy related gene. Importantly, sleep disturbances prior to IFN treatment is a risk factor for treatment-emergent depression. Treatment of sleep problems in HCV consists of sleep hygiene, sleep-promoting agents such as antihistamines, probenzodiazepines (such as zolpidem, zaleplon), and sedative antidepressants (trazodone, mirtazapine) for insomnia, modafinil for hypersomnia, and gabapentin and iron replacement for restless leg syndrome (57, 58).

### 3.5. Neuropsychological Dysfunction

Several studies have reported evidence of cognitive dysfunction in patients with HCV. Although cognitive dysfunction is seen in hepatic encephalopathy as well, the nature of the condition is different from what is seen in patients with HCV without hepatic encephalopathy. Many patients with HCV suffer from impaired concentration and memory, a condition which is called “brain fog” (59). Impairment of concentration, sustained attention, attentional processing, working memory, and visuomotor processing speed have been reported in HCV-positive patients (15, 18, 60, 61). A recent interesting study has provided evidence for decision-making dysfunction in patients with HCV with a bias toward smaller immediate rewards rather than larger delayed rewards (62). In general, constructional abilities and non-verbal recall is intact in these patients (60). Electrophysiological studies have also shown evidence of cognitive dysfunction in HCV positive patients (19, 63). Prepulse inhibition (PPI) which reflects the function of forebrain cortico-striatial-pallidal-thalamic circuit maybe a useful and sensitive measure of attentional processing deficits in HCV patients (64). Moreover, comparison of HCV with HBV infected patients has shown poorer visuo-spatial memory performance in HCV subjects (65). Studies in specific populations of HCV patients such as women, children, and those without substance abuse history (66, 67) have also demonstrated evidence of cognitive dysfunction in these patients. In a review, Perry et al. (67) evaluated four possible mechanisms for cognitive dysfunction in patients with HCV. Premorbid characteristics and habits (particularly substance abuse) could not explain the cognitive dysfunction in these patients, because patients without such habits still showed various levels of cognitive impairment. Moreover, psychiatric disorders were not associated with cognitive problems in most of the studies. A third explanation is virus invasion of the brain, which although present does not contribute very much to the process of cognitive dysfunction. The last explanation given by Perry and colleagues is the activation of the brain inflammatory system by the virus. In addition to these explanations, the current study group has recently shown reduction of BDNF levels in the serum of these patients (67). Although the cognitive processes of the patients were not studied, it was shown that fatigue (which is usually of central type in patients with HCV) was inversely related to BDNF levels. Although no one has studied the relation between cognition and BDNF in the patients with HCV, an association between the two seems likely (11). Furthermore, two important factors that might exacerbate cognitive impairment of HCV patients are co-infection with HIV and INF (but not low dose peg interferon) treatment (64, 68). In a study, following treatment with INF/ribavirin, the patients who were classified as SVR showed reduced brain inflammation/infection and improved neuropsychological function compared with the patients who were non-responders (69).

### 3.6. Neurological Involvement

Neurological complications of HCV infection are uncommon. In a review, Monaco et al. (70) identified various types of CNS involvement in HCV-infected individuals. These included acute cerebrovascular accidents, CNS vasculitis, encephalopathic syndromes, white matter involvement, inflammatory disorders of CNS, and peripheral neuropathy (70).

### 3.7. Evidence of Brain Involvement

Substantial evidence of reduced mental and physical health and impaired cognition has led to this hypothesis that HCV can affect brain function. The brain dysfunction in HCV is largely unrelated to substance abuse, INF treatment, disease severity, and hepatic encephalopathy (71). There are several lines of evidence which suggest brain changes in HCV-positive patients. Neuroimaging studies have shown strong evidence for brain alterations particularly of basal ganglia in patients with HCV (46). Moreover, both dopaminergic and serotoninergic dysfunctions have been demonstrated in the brain of HCV-infect individuals (72).Fletcher and McKeating reviewed the evidence on the HCV involvement of the brain. PCR-based methods provided evidence for the presence of HCV RNA (including negative-strand intermediate which has replicative role) in the brain of individuals with HCV infection. Moreover, both neuroimaging and post-mortem studies have shown evidence of microglial and inflammatory activation in patients with HCV (59).

### 3.8. Effect of HIV-HCV Comorbidity on Psychosocial Aspects of Life

Substance abuse and HIV-HCV co-infection frequently concur in a single patient. All of these conditions can have deleterious effect on psychosocial health of the patients. Martin-Thormeyer et al.(73) have reviewed the impact of these comorbidities on the psychosocial health of the patients. Because of similar routes of transmission, up to 90% of patients might have con-infection according to some European series. HRQOL of the HIV-HCV co-infected individuals is poorer than those of HIV-infected or HIV-HBV co-infected individuals according to the review. Depression, fatigue, and substance abuse are important predictors of poorer HRQOL in HIV-HCV co-infected patients. Decreased processing and psychomotor speed in co-infected individuals compared with those of mono-infected individuals have been reported in several studies (33). However not all studies showed poorer cognitive functioning in HIV-HCV co-infected individuals including a large study on more than 1000 women (74).

### 3.9. Psychosocial Experience of Living with HCV

In a narrative systematic review, Miller et al. (7) identified 43 studies on this subject, all of which had been conducted in Western countries. HCV diagnosis was found to have deteriorating effects on social functioning in most of the studies. The authors also concluded that HCV was associated with social marginalization, impairment of intimate and family relationships, reduction in substance and alcohol abuse, changes in dietary intake, reduced sense of well-being due to fear of transmission and prognosis, fatigue, hopelessness, depression, anger, and stigma (7).

### 3.10. Anger and Irritability

Anger and irritability are important but underestimated side effects of INF treatment. In a review of ten studies on the effect of antiviral treatment on irritability, up to 75% of the patients experienced irritability following treatment (75). Irritability might be secondary to other mood disorders which often arise during antiviral treatment. Anger/irritability during antiviral treatment has been linked to TNF-alpha polymorphisms but not serotonin transporter polymorphism (76). Prolactin was also associated with anger in HCV patients in one study (77).

### 3.11. Stigma

In a concept analysis, stigma in the context of HCV was defined as a subjective and variable, perceived (usually) negative phenomenon. From this point of view, stigma has interrelated intrapersonal, interpersonal and structural dimensions (78). A distinctive aspect of the HCV-related stigma is its relation to IDU (78). Stigma negatively affects the HRQOL, mental health, and social life of the patients, and leads to difficulties with receiving or accepting treatment (79, 80). Poor social and work adjustment, lower acceptance of the illness, and higher subjective complaints are other problems associated with stigmatization (81). Studies showed that women generally experienced more stigmatization (80, 82, 83). In different series from 35% to more than 85% of the patients with HCV were reported to experience stigma (80, 84, 85). Stigma can occur as a result of discrimination. In an Australian study on over 500 patients with HCV, 65% of the patients reported discrimination which most commonly had occurred in the healthcare setting (101).

### 3.12. Coping

Coping is the individual cognitive and behavioral response to perceived stress. In a study on over 100 individuals with HCV, the most commonly used coping style was problem-solving behavior followed by distraction and self-revalorization, religiousness and search for meaning, cognitive avoidance and dissimulation, and depressive coping. Importantly, recent diagnosis was associated with the highest levels of problem-solving behavior and the lowest levels of depression. Other factors such as mode of acquisition were found unlikely to affect the coping styles used (86). Several studies have reported using inappropriate coping strategies in patients with HCV which may negatively affect several aspects of their management (87-89).

### 3.13. Social Support

Social support is an important aspect of the lives of the HCV-infected individuals. In a study of 340 patients with HCV infection, 45% noted the loss of at least one relationship following diagnosis of infection (90). Studies have shown evidence of low social support in HCV-infected patients and its association with living alone, being unemployed, poorer HRQOL, exclusion from antiviral therapy, physical symptoms, psychiatric comorbidities, and IDU (65, 90-93). Therefore, support groups may improve health outcomes in patients living with chronic HCV infection (94).

### 3.14. Psychosocial Barriers to the Treatment 

Providing care to the patients with HCV is a complex process because of characteristics of the patients, the disease, health care workers, and the system (95, 96). In a study, two third of the HCV patients declined treatment because of asymptomatic nature of their infection together with concerns regarding treatment side effects (97). In over one third of the patients with HCV who do not receive treatment the reason is psychosocial (98). An important aspect of the care for these patients comprises management of mental health problems and in particular intravenous drugs use (IDU). The patients who abuse substance should be treated with patience and tolerance in a mutual and trusting relationship with their provider. Many patients have had negative experiences with judgmental and unresponsive health-care system, and therefore must be treated in a respectful, nonjudgmental, and supportive manner (95). Poverty, fear of arrest, social isolation, involvement in unstable primary care relationship, discrimination, several comorbidities, unavailability of expertise, and high expenses of the management all add to the complexity of care in these patients (79, 95, 96). Moreover, physicians frequently attribute the treatment failure to drug addiction, and thus many are reluctant to see addicted patients (99). A client-centered approach in a multidisciplinary team is probably the best way to increase the rate of treatment satisfaction and thus adherence in HCV patients with drug addiction (95). Edlin et al. (95) reviewed principals of care in these patients. An issue of raising concern is stopping or withdrawing optimal treatment of HCV because of psychiatric complications. In a retrospective study conducted in Minnesota, USA 101 physicians who were treating patients with chronic HCV were interviewed. One in five patients had not received optimal management of HCV due to a psychiatric problem, and less than half of the physicians were collaborating with psychiatry or psychology experts (100). This rate is probably much higher in the setting of crowded HCV clinics in third world countries. On the other hand, it has been shown that patients with optimal psychiatric management adhere more strictly to their antiviral regimen than those patients without (101). Considering several studies together, Loftis et al.(96) have shown that little evidence supports withholding treatment from eligible patients with HCV and comorbid substance abuse (other than alcohol) or psychiatric disorders. However, these patients should be closely monitored for psychiatric side effects of treatment and be actively treated for their mental illness (96).

## 4. Conclusions

Patients with HCV infection had complex neuropsychiatric and psychosocial problems. These problems are challenges for management of HCV infection, affect the patient’s care significantly, and might alter the course of the disease. A multidisciplinary approach, a supportive environment, and a nonjudgmental healthcare team are required for optimal medical and psychosocial management of patients with HCV.
